# Prevalence of Antimicrobial Resistant *Escherichia coli* from Sinking Creek in Northeast Tennessee

**DOI:** 10.3390/ijerph21101285

**Published:** 2024-09-26

**Authors:** Walid Q. Alali, Phillip Scheuerman, Clara McClure, Achala Ghimire, Priscilla Owusu-Mensah, Jacob Schultz, Timothy Andrew Joyner

**Affiliations:** 1 Department of Biostatistics & Epidemiology, College of Public Health, East Tennessee State University, Johnson City, TN 37614, USA; ghimirea@etsu.edu; 2 Department of Environmental and Occupational Health and Safety Sciences, College of Public Health, East Tennessee State University, Johnson City, TN 37614, USA; philsche@etsu.edu (P.S.); mcclurec@etsu.edu (C.M.); owusumensah@etsu.edu (P.O.-M.); jacob.l.schultz70@gmail.com (J.S.); 3 Department of Geosciences, College of Arts and Sciences, East Tennessee State University, Johnson City, TN 37614, USA; joynert@etsu.edu

**Keywords:** antimicrobial resistance, *Escherichia coli*, surface water, multidrug resistance, colistin, public health, environmental contamination

## Abstract

Antibiotic resistance (AR) is a critical global health threat exacerbated by complex human–animal–environment interactions. Aquatic environments, particularly surface water systems, can serve as reservoirs and transmission routes for AR bacteria. This study investigated the prevalence of AR *E. coli* in Sinking Creek, a pathogen-impacted creek in Northeast Tennessee. Water samples were collected monthly from four sites along the creek over a 6-month period. *E. coli* isolates were cultured, identified, and tested for susceptibility to eight antibiotics using the Kirby–Bauer disk diffusion method and broth disk elution method for colistin. Data were analyzed to determine the prevalence of AR and multidrug resistance (MDR) among isolates. Of the 122 water samples, 89.3% contained *E. coli*. Among the 177 isolates tested, resistance was highest to ciprofloxacin (64.2%) and nitrofurantoin (62.7%), and lowest to fosfomycin (14.1%) and colistin (6.0%). Significant differences in resistance to ceftriaxone and amoxicillin/clavulanic acid were observed between sampling sites. MDR was prevalent in 47.5% of isolates, with 5.1% resistant to seven antibiotics. The most frequent MDR patterns (6.8%) included three antibiotics: ceftriaxone, ciprofloxacin, and nitrofurantoin. The high prevalence of AR *E. coli* in Sinking Creek poses a significant public health risk, highlighting the need for ongoing surveillance and intervention strategies to prevent the spread of AR bacteria.

## 1. Introduction

Antibiotic resistance (AR) poses a significant global health threat and has been recognized by the WHO as one of the top ten threats to human health [1,2]. The epidemiology of AR bacteria is quite complex due to the human–animal–environmental interaction involved in developing and transmitting AR organisms [3]. Aquatic environments are increasingly recognized as reservoirs and pathways for the transmission of AR [4,5,6,7]. Aquatic ecosystems, particularly surface water systems, are exposed to pollutants from various anthropogenic and agricultural sources [8,9]. Surface water continuously receives both non-pathogenic and pathogenic bacteria from various sources such as septic systems, wastewater treatment plants, agriculture, and wildlife [10,11]. Antibiotic-resistant bacteria in aquatic environment can present a health risk to humans due to drinking contaminated water, ingested contaminated water during recreational activities, or/and the consumption of produce irrigated with contaminated water [4,12,13,14,15].

*Escherichia coli*, which are abundant in the gastrointestinal tract of humans and animals, can act as carriers or reservoirs of AR genes [16,17]. They are also capable of exchanging AR genetic material with bacteria of the same or different bacterial species [18,19]. Studies have demonstrated that *E. coli* is a very adaptable organism capable of thriving in external environments, including surface waters [20,21]. Specifically, research has reported the ubiquitous presence of AR *E. coli* in various water sources and the emergence of multi-drug resistant *E. coli* [22,23]. A previous study conducted in Upper Oconee Creek, a mixed-use watershed in Athens, GA, USA, revealed that 6.9% (*n* = 496 *E. coli* isolates from 458 water samples) exhibited resistance to at least 1 of the 14 tested antibiotics, while 3% of the isolates were resistant to multiple antibiotics [24].

Antibiotic resistant *E. coli* has been detected in various water sources, including surface water, drinking water, recreational water, and wastewater. For instance, in the Chesapeake Bay watershed, *E. coli* isolates, detected in streams and rivers, particularly in areas with heavy livestock farming, were pan-susceptible (67%), resistant to at least one antibiotic (33%), resistant to ampicillin (24%), resistant to cefazolin (13%), and 8% were multi-drug-resistant [25]. In another study conducted in Colorado, the authors showed that the prevalence of *E. coli* isolates exhibiting ESBL resistance increased by 1.70% in receiving surface water compared to the wastewater effluent [26]. In Iowa, authors reported that *E. coli* isolates were resistant to ESBL (44%), cephalosporin (42%), and carbapenems (6%) in the water samples obtained from 24 stream locations across the state [27].

Despite these studies on *E. coli* from diverse aquatic ecosystems, those focused on AR *E. coli* and in anthropogenic-impacted aquatic ecosystems remain scarce, particularly in developed countries. A study conducted on an anthropogenic-impacted stream in Indiana [28] showed that 31.3% of *E. coli* O157:H7 were resistant to tetracycline, and 3.1% were resistant to ciprofloxacin. In developing countries, a review article from South America highlighted the health and environmental issues with antibiotic resistance in anthropogenic-impacted water systems [29]. Moreover, studies from Asia, such as in Vietnam and Malaysia, reported various AR diversities in anthropogenic-impacted aquatic systems [30,31].

Previous research on Sinking Creek (an anthropogenic-impacted aquatic ecosystem in Northeast Tennessee) has shown high levels of fecal contamination using coliforms including *E. coli* as water quality indicators [32,33,34,35]. These research studies revealed the relationship between several environmental factors, such as the chemical and microbial parameters of water and *E. coli* concentrations in Sinking Creek. Additionally, McCurdy et al. [36] indicated that sources of *E. coli* in Sinking Creek might be contamination from residential wastewater. However, none of these studies reported AR levels in this creek. Therefore, this study aimed to establish a baseline prevalence of AR *E. coli* in water samples from Sinking Creek.

## 2. Materials and Methods

### 2.1. Study Area and Sample Collection

**Figure 1:**
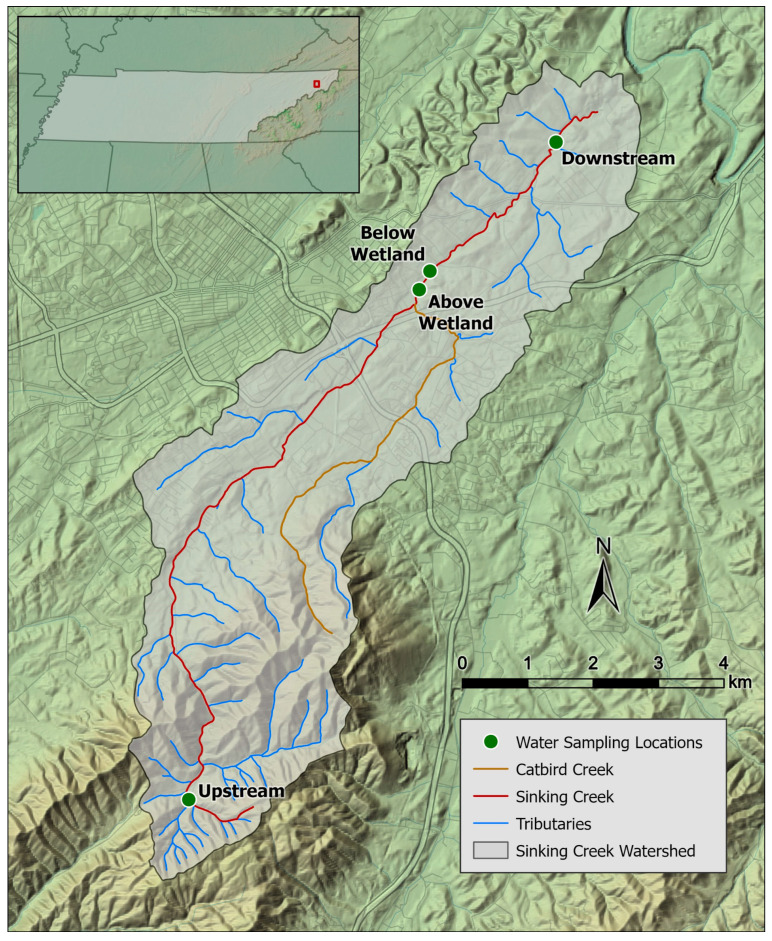
Water sampling site along Sinking Creek in Northeast Tennessee. Red box indicates the location of Sinking Creek in Northeast Tennessee.

Sinking Creek was our study site. This creek is part of the Watauga River watershed (HUC 06010103), which is located in multiple counties in Eastern Tennessee. Sinking Creek land use is a mixture of forest (79.8%), agricultural (9.8%), and urban (5%), but is continually changing due to rapid economic and housing development [37]. This creek has 10 stream miles that do not meet the USEPA standard for *E. coli* [37]. Four sampling points were selected along the creek (Figure 1) based on previous *E. coli* concentration data [32,34,35]. Sinking Creek flows southwest to northeast, starting in the mountainous area south of Johnson City, TN, USA, and submerges underground northeast of Johnson City before entering the Watauga River. One sampling point was upstream near the headwaters, one was upstream of a wetland (i.e., above wetland) in Jacob’s Nature Park (Johnson City, TN, USA), one was downstream of the wetland (i.e., below wetland), and one was further downstream just above where the creek submerges underground (Figure 1). Sinking Creek flows through the wetland, and during the development of Jacob’s Nature Park, the wetland was expanded to reduce the *E. coli* in Sinking Creek. Ongoing monitoring studies have shown minimal reduction in *E. coli* [32,33,34,35].

At each sampling point, 3–4 water samples (15 mL each) were collected from the middle of the creek using a sterile 15-mL Falcon tube (Fisher Scientific, Pittsburgh, PA, USA). Samples were collected monthly over 6 months (June–December 2023). All samples were transported to the ETSU-Environmental Health Science Laboratory (EHSL) on ice, and the analyses were initiated within 3 h of arrival.

### 2.2. Microbiological Analyses

#### 2.2.1. *E. coli* Isolation

Upon arrival at the EHSL, the water samples were cultured to isolate *E. coli*. The samples were vortexed and then filtered using 25 mm, 0.45 µm pore polycarbonate nucleopore filters (Osmonics, Inc., Minnetonka, MN, USA) using a vacuum pump according to the USEPA method 1603 [38]. After filtration, the filters were placed on CHROMagar™ *E. coli* (Difco, Becton Dickenson, Sparks, MD, USA) plates and incubated for 16–18 h at 35 °C. After incubation, up to three typical colonies (blueish color with smooth surface) were subsequently streaked onto CHROMagar plates and incubated 16–18 h at 35 °C, to isolate pure colonies. The colonies were screened for lactose fermentation using the Indole spot test (Remel, Thermoscientific, Lenexa, KS, USA). Biochemical test strips (RapID ONE test; Remel, Lenexa, KS, USA) were used as quality control on every 25th isolate to confirm that the isolated bacteria were *E. coli*. The isolates were transferred into Cryovial tubes (Fisher Scientific, Pittsburgh, PA, USA) and stored at −80 °C until further analysis.

#### 2.2.2. Antibiotic Susceptibility Testing

Antibiotic susceptibility testing was carried out using the Kirby–Bauer disk diffusion method. About 3–4 *E. coli* colonies were picked from a CHROMagar™ *E. coli* plate, inoculated into 2 mL phosphate buffered saline (PBS) solution, then vortexed to achieve an inoculum equivalent to the 0.5 McFarland standard. One millimeter aliquot from the *E. coli*-PBS suspension was transferred into 4 mL of Muller Hinton (MH) (Fisher Scientific, Pittsburgh, PA, USA) soft agar and vortexed. This solution was poured over an MH agar plate to form a thin overlay agar. The plates were left for 10 min to solidify before an antibiotic disk placement. The disks were placed on the MH agar plates using an antibiotic disk dispenser (Fisher Scientific, Pittsburgh, PA, USA). The seven antibiotic disks used in this study included the following: amoxicillin/clavulanic acid, tetracycline, trimethoprim-sulfamethoxazole, ceftriaxone, ciprofloxacin, nitrofurantoin, and fosfomycin (Table 1). The plates were placed agar-side up in the incubator for 16–18 h at 35 °C. Thereafter, the diameters of zones of inhibition for each antibiotic were recorded in mm using an electronic caliper. These measurements were interpreted as susceptible, intermediate, or resistant according to the Clinical and Laboratory Standards Institute (CLSI) guidelines [39]. *E. coli* ATCC 25922 (American Type Culture Collection, Manassas, VA, USA) strain was used as a reference strain. The seven antibiotics used to test for *E. coli* isolates antibiotic susceptibility and their breakpoints are shown in Table 1. For data analyses, intermediate antibiotic susceptibility test results were reclassified as susceptible.

#### 2.2.3. Polymyxin (Colistin) Susceptibility Testing

Antimicrobial susceptibility testing of colistin was performed using the broth disk elution method as per CLSI recommendations [39]. Briefly, frozen *E. coli* isolates were revived from the Cryovials on blood agar plates, then 3–5 *E. coli* isolates were inoculated into sterile saline (5 mL) and vortexed to attain an inoculum corresponding to 0.5 McFarland standard. Colistin disks at three different concentrations 1, 2, and 4 µg, were added to three Cation-Adjusted Mueller–Hinton Broth (CAMHB) tubes, then gently vortexed and left for at least 30 min at room temperature. An additional CAMHB without colistin was used as a negative control. An inoculum of 50 µL from the *E. coli* suspension was added to each CAMHB tube, vortexed, and incubated at 35 °C for 16–20 h. For inoculum purity check, a 10 µL loop from the *E. coli* suspension was subcultured onto blood agar plates and incubated similarly to the CAMHB tubes. Thereafter, the MIC was read as the lowest concentration where the growth of the tested isolate was completely inhibited. The results were interpreted as susceptible or intermediate if ≤2 µg/mL or resistant if ≥4 if µg/mL. A positive control ATCC *E. coli* 25922 was used as a colistin reference strain. We were able to revive 50 frozen isolates out of 177 for colistin testing.

### 2.3. Data Analysis

The antibiotic resistance outcomes (resistant or susceptible) and multidrug-resistant (MDR) isolates (defined as resistant to at least an antibiotic in three different antimicrobial classes) were cross-tabulated by sampling the location. A Fisher’s exact test or likelihood ratio chi-square test, as appropriate, was used for the analysis, conducted in STATA version 17.0 software (Stata Corp., College Station, TX, USA).

## 3. Results

Of the 122 water samples tested, 89.3% had at least one *E. coli*-positive isolate. There was no significant difference in *E. coli* isolation percentages between the sampling sites. A total of 177 isolates were tested for antibiotic susceptibility. The highest prevalence of AR *E. coli* to individual antibiotics was to ciprofloxacin (64.2%) and nitrofurantoin (62.7%) and the lowest was to fosfomycin (14.1%) and colistin (6.0%) (Table 2). There were significant (*p* < 0.05) differences between sampling sites for *E. coli* resistance to ceftriaxone and amoxicillin/clavulanic acid, with the highest prevalence in wetland locations. The overall individual AR to colistin was 6% out of 50 isolates tested. Three out of 50 isolates had growth at 4 µg/mL colistin concentration.

Multidrug resistance was common among *E. coli* isolates (i.e., 47.5%) with 5.1% of the 177 isolates being resistant to seven antibiotics except for colistin (Table 3). One of the most common MDR patterns was ceftriaxone, ciprofloxacin, nitrofurantoin (7.5%), ceftriaxone, tetracycline, ciprofloxacin, nitrofurantoin (6.8%), and ceftriaxone, tetracycline, ciprofloxacin, amoxicillin/clavulanic acid, fosfomycin, trimethoprim-sulfamethoxazole, nitrofurantoin (6.1%). Multi-drug-resistant isolates that included colistin also included nitrofurantoin, amoxicillin/clavulanic acid, tetracycline, and/or ciprofloxacin.

**Figure 2:**
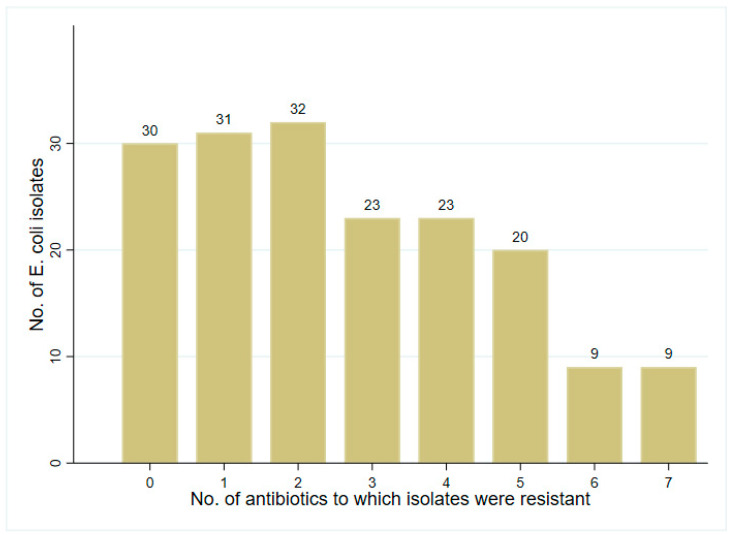
Bar chart showing the frequency distribution of phenotypic antibiotic resistance to up to eight antibiotics among *E. coli* isolates (N = 177) from water samples originating from Sinking Creek in Northeast Tennessee.

The frequency distribution of the antibiotic-resistant phenotypes to the eight antibiotics is shown in Figure 2. Nearly 23 isolates (13%) were resistant to three antibiotics as well as 23 (13%) to four antibiotics. Furthermore, 20 isolates (11.3%), 9 isolates (5.1%), and 9 isolates (5.1%) were resistant to five, six, and seven antibiotics, respectively. The distribution of the prevalence and phenotype of MDR *E. coli* by sampling site is shown in Table 4. The highest MDR AR *E. coli* prevalence occurred in samples collected from the wetland sampling sites. The most frequent MDR patterns (6.8%) included three antibiotics: ceftriaxone, ciprofloxacin, and nitrofurantoin, regardless of the site. While the highest MDR prevalence (17.9%) was in isolates from the downstream site (ceftriaxone, ciprofloxacin, and nitrofurantoin), the MDR in isolates from the upstream site had four different phenotypes.

## 4. Discussion

Our study established a baseline prevalence of AR *E. coli* from a pathogen-impacted creek in Northeast Tennessee. While previous research conducted on the same stream showed that it exceeded USEPA standards for coliform bacteria including *E. coli* [32,33,34,35,36], the prevalence of AR was not quantified. Our study demonstrates a high prevalence of MDR *E. coli* present in this creek, which is a significant public health concern because of the potential dissemination of these isolates to humans through drinking water and recreational activities [40]. Sinking Creek flows through multiple recreation areas such as Jacob’s Nature Park (Johnson City, TN, USA), residential areas in two counties (Carter and Washington), and discharges in Watauga River, which is used for recreational activities. Previous studies have reported the possible human exposure to AR *E. coli* through recreational activities [41,42].

Land use in the Sinking Creek watershed includes a mixture of agriculture and urban, which could contribute to AR *E. coli* in the water [32,35]. Previous reports showed that defective septic tanks from residential areas can overflow or leak into nearby water bodies [43,44,45]. The high prevalence of resistance to ciprofloxacin (64.2%), nitrofurantoin (62.7%), and ceftriaxone (40.1%) in this study, which are antibiotics used exclusively in human medicine [46], might indicate human contamination sources. A previous study conducted in a mixed-use watershed in Georgia revealed that 6.5% of *E. coli* isolates were resistant to at least one antibiotic such as tetracycline (4.1%), and 3% were resistant to multiple antibiotics [24]. These data are lower than 30.5% of *E. coli* in this study exhibiting tetracycline resistance. This difference might be due to differences in land use in this region and other environmental exposure factors. The mixture of land use around Sinking Creek makes it a valuable representative for other watersheds of a similar size.

Multidrug resistance was common among *E. coli* isolates (i.e., 47.5%) with most frequent MDR patterns (6.8%) including ceftriaxone, ciprofloxacin, and nitrofurantoin. Ciprofloxacin and nitrofurantoin are frequently used to treat urinary tract infections (UTIs) [46]. Several reports have shown that *E. coli* isolates resistant to ciprofloxacin and nitrofurantoin were common causes of UTIs [47,48]. The highest MDR AR *E. coli* prevalence was in samples collected from the wetland sampling sites. These sites are located within Jacob’s Nature Park. Furthermore, we observed that water flows slower in these sites than upstream and downstream sites. The increased input of organic matter from the wetland in the middle section of Sinking Creek may also influence bacterial growth [32,33,34,35,49]. Moreover, agriculture pollution, such as water runoff, can also be a source of AR *E. coli* contamination, as has been shown in a previous study [50]. Previous studies on surface water (streams, rivers, and lakes) showed variability in AR *E. coli* prevalence. For instance, a high AR *E. coli* prevalence was reported from urban rivers in Milwaukee, Wisconsin, where all isolates were determined to be multi-drug-resistant [51]. Resistance to tetracycline (35%), ciprofloxacin (65%), trimethoprim (50%), sulfamethoxazole (88%), and fosfomycin (55%) was also reported, and is higher than what is reported in this study. The authors explained that their findings were due to river contamination with wastewater effluent containing a greater incidence of AR *E. coli*, history of contamination from human activities, and historical high abundance of antibiotics in the river [51]. A study conducted at rivers in South Carolina [52] revealed that 47% of *E. coli* isolates were resistant to multiple antibiotics, whereas a study conducted at a pathogen-impacted stream in Indiana [28] that tested 21 *E. coli* O157:H7 isolates showed that 31.3% were resistant to tetracycline (similar to our findings), but 3.1% were resistant to ciprofloxacin compared to 64.2% in this study. Another study reported a high prevalence (i.e., 63%) of multi-drug-resistant *E. coli* isolates from multiple water sources including rivers and streams in Ghana which might be due to human and agriculture non-point source contamination [53]. Moreover, resistance to ciprofloxacin (74.2%) and tetracycline (21.5%) was relatively high in their study, in agreement with our data. This variability in study findings could be attributed to factors such as historically pathogen-impacted water, land use and potential sources of contamination, the number of samples/isolates tested, and choices of antibiotics used for susceptibility testing.

Our study revealed that among 50 *E. coli* isolates tested for colistin resistance, 3 (6%) were resistant. The detection of colistin resistance in *E. coli* isolates from surface water that is pathogen-impaired is a significant finding which may explain the detection of this resistance phenotype. Few studies in the U.S. have reported colistin resistance in *E. coli* originating from surface water. One study conducted at a watershed in Indiana reported colistin resistance in one *E. coli* isolate out of 70 tested [54]. The authors did not provide an explanation for colistin resistance. At another watershed in Indiana, the authors did not detect colistin resistance among 97 *E. coli* isolates [55]. Nonetheless, colistin resistance in studies conducted in developing countries such as Thailand and China was not uncommon [56,57]. Colistin resistance has been identified in humans as well as in animals and their products in the U.S. Specifically, *E. coli* resistance to colistin was previously detected at a low level; 0.1% in animals at slaughter and 0.02% from animal meat [58,59] and in a few patients in the U.S. [60,61]. A recent report from Tennessee revealed that raw milk contained four E. coli isolates with the *pmrB* gene, which is commonly associated with colistin resistance [62]. There are global concerns over the rise in bacterial resistance to colistin [63]. This antibiotic is often considered a “last-resort” drug for treating infections with Gram-negative bacteria that exhibit resistance to multiple antibiotics, such as those caused by AR *E. coli*. When bacteria resist colistin, treatment options for severe infections become extremely limited, increasing the risk of treatment failure and mortality.

There are limitations to this study. First, it was conducted on a single pathogen-impacted stream in Northeast Tennessee, which may not be representative of other rivers in the region. However, this is the first antibiotic resistance study conducted on a stream in this region and demonstrates the potential negative impact on human health. Additionally, the study could be a representative for other watersheds of similar size and environment. Second, the study was conducted over a 6-month period, so seasonality could not be assessed. Our primary goal was to determine the prevalence of AR *E. coli*, and we plan to follow up with a longitudinal study to assess seasonality among other risk factors. Third, factors such as water chemistry (pH, total dissolved solids), stream depth, width, and velocity were not consistently measured during each sampling visit and thus were not included in the analysis. Since we aimed to establish a baseline prevalence of AR *E. coli* in water samples, these factors might have had a limited impact on AR phenotypes per *E. coli* isolated from the samples.

## 5. Conclusions

In conclusion, our study has established a baseline prevalence of AR *E. coli* in Sinking Creek, a pathogen-impacted river in Northeast Tennessee. The high prevalence (i.e., 47.5%) of MDR *E. coli*, particularly those resistant to ciprofloxacin (64.2%), nitrofurantoin (62.7%), and ceftriaxone (40.1%), drugs used in treatment of a variety of infections, underscores a significant public health concern.

The land use in the Sinking Creek watershed includes a mixture of agriculture and urban and could be a contributing source of AR *E. coli* in the water. The detection of colistin-resistant *E. coli* (6%) is a concern to human and environmental health given the prior low detection levels in the U.S. in humans, animals, and the environment.

This study emphasizes the need for continuous monitoring of environmental dissemination and sources of AR bacteria to be able to mitigate the spread of resistant bacteria from aquatic ecosystems to humans. Further longitudinal studies are warranted to explore seasonal variations and other risk factors influencing the prevalence of AR *E. coli* in this region.

## Figures and Tables

**Table 1:** The disk diffusion zone of inhibition interpretation for antibiotics used to test susceptibility of *E. coli* isolates (N = 177) from water samples of Sinking Creek (Johnson City, TN, USA).

Drug Class/Antibiotic ^1^	Concentration (μg)	Zones of Inhibition Diameter in mm Interpretation ^2^		
		S	I	R
Sulfonamides				
Trimethoprim & Sulfamethoxazole	1.25/23.75	≥16	45,245	≤10
Penicillin β-lactamase inhibitor combinations				
Amoxicillin/ Clavulanic acid	20/10	≥18	14–17	≤13
Tetracyclines				
Tetracycline	30	≥15	45,274	≤11
Cephalosporins				
Ceftriaxone	30	≥23	20–22	≤19
Fluoroquinolone				
Ciprofloxacin	5	≥31	21–30	≤20
Urinary anti-infectives				
Nitrofurantoin	300	≥17	15–16	≤14
Phosphonic antibiotics				
Fosfomycin	200	≥16	13–15	≤12

^1^ Colistin susceptibility testing was performed according to the Clinical and Laboratory Standards Institute (CLSI) broth disk elution method using three concentrations (1, 2, and 4 µg). The results were interpreted as susceptible or intermediate ≤ 2 µg/mL or resistant if ≥4 if µg/mL. ^2^ The zones of inhibition values interpretation into sensitive (S), intermediate (I), and resistant (R) was according to the CLSI guidelines.

**Table 2:** Prevalence of antibiotic-resistant *E. coli* isolates (N = 177) recovered from water samples from Sinking Creek, Johnson City, TN, USA.

	No. (%) of Isolates					
Antibiotics/Sampling Site	Upstream (*n* = 63)	Above Wetland (*n* = 43)	Below Wetland (*n* = 29)	Downstream (*n* = 42)	*p*-Value ^1^	Overall Prevalence (*n* = 177)
Ceftriaxone	23 (36.5)	23 (53.5)	16 (55.2)	9 (21.4)	0.005	71 (40.1)
Tetracycline	17 (27.0)	14 (32.6)	12 (41.4)	11 (26.2)	0.482	54 (30.5)
Ciprofloxacin	43 (68.3)	28 (65.1)	15 (51.7)	27 (64.3)	0.488	113 (63.8)
Amoxicillin/ Clavulanic acid	18 (28.6)	19 (44.2)	10 (34.5)	5 (11.9)	0.008	52 (29.4)
Fosfomycin	12 (19.1)	9 (20.9)	2 (6.9)	2 (4.8)	0.064	25 (14.1)
Trimethoprim-Sulfamethoxazole	14 (22.2)	12 (27.9)	8 (27.6)	4 (9.5)	0.126	38 (21.5)
Nitrofurantoin	34 (54.0)	30 (69.8)	20 (69.0)	27 (64.3)	0.333	111 (62.7)
Colistin ^2^	1 (7.1)	0 (0)	2 (18.1)	0 (0.0)	0.243	3 (6.0)

^1^ *p*-value < 0.05 indicates that percentages of antibiotic-resistant *E. coli* isolates were significantly different by sampling sites based on chi-square test in STATA 17.0 software; ^2^ For colistin, the number of isolates tested from upstream, above wetland, below wetland, and downstream were 14, 11, 11, and 14, respectively.

**Table 3:** Antibiotic-resistant phenotypes of *E. coli* isolates (N = 177) from water samples collected from Sinking Creek, Johnson City, TN, USA.

Resistant Phenotype ^1^	Frequency	Percentage
No resistance	30	17.00
Resistant to only one antibiotic	31	17.50
CIP_NIF	20	13.61
CIP	13	8.84
NIF_	11	7.48
CRO_CIP_NIF	10	6.80
CRO_TET_CIP_NIF	10	6.80
CRO_TET_CIP_AMC_FOS_SXT_NIF	9	6.12
COL	3	6.00
TET_CIP	6	4.08
CRO_CIP_AMC_NIF	5	3.40
CRO_TET_CIP_AMC_NIF	5	3.40
CRO_CIP_AMC_FOS_SXT_NIF	4	2.72
CRO_CIP_AMC_SXT_NIF	4	2.72
CRO_TET_CIP_SXT_NIF	4	2.72
TET	4	2.72
CRO_AMC_FOS_SXT_NIF	3	2.04
CRO_TET_CIP_AMC_SXT_NIF	3	2.04
CIP_AMC_NIF	2	1.36
CIP_SXT	2	1.36
CRO_CIP	2	1.36
CRO_TET_AMC_SXT_NIF	2	1.36
TET_CIP_NIF	2	1.36
AMC	1	0.68
AMC_NIF	1	0.68
AMC_NIF_COL	1	0.68
CIP_AMC	1	0.68
CIP_AMC_FOS_SXT_NIF	1	0.68
CIP_AMC_SXT_NIF	1	0.68
CRO_AMC_NIF	1	0.68
CRO_AMC_SXT_NIF	1	0.68
CRO_CIP_AMC_FOS_NIF	1	0.68
CRO_CIP_FOS_NIF	1	0.68
CRO_CIP_FOS_SXT_NIF	1	0.68
CRO_FOS_NIF	1	0.68
CRO_TET_AMC_FOS_SXT_NIF	1	0.68
CRO_TET_CIP	1	0.68
CRO_TET_CIP_AMC_FOS_NIF	1	0.68
CRO_TET_SXT_NIF	1	0.68
FOS_NIF	1	0.68
TET_AMC_NIF	1	0.68
TET_AMC_NIF_COL	1	0.68
TET_CIP_AMC	1	0.68
TET_CIP_AMC_FOS	1	0.68
TET_CIP_COL	1	0.68
TET_CIP_SXT_NIF	1	0.68

**^1^** T CIP, Ciprofloxacin; NIF, Nitrofurantoin; CRO, Ceftriaxone; TET, Tetracycline; AMC, Amoxicillin/Clavulanic acid; COL, Colistin; SXT, Trimethoprim-Sulfamethoxazole; FOS, Fosfomycin.

**Table 4:** Distribution of antibiotic-resistant *E. coli* isolates from water samples by sampling site (Sinking creek, Johnson City, TN, USA) ^1^.

Sampling Site (No. Isolates)	MDR No. (%)	Top Most Frequent MDR (>5%) Phenotype and %	Highest MDR Profile and %
Upstream (*n* = 63)	27 (42.9)	CRO_TET_CIP_AMC_FOS_SXT_NIF (5.7%) CRO_CIP_AMC_FOS_SXT_NIF (5.7%) CRO_AMC_FOS_SXT_NIF (5.7%) CRO_CIP_NIF (5.7%)	CRO_TET_CIP_AMC_FOS_SXT_NIF (5.7%)
Above Wetland (*n* = 43)	26 (60.7)	CRO_CIP_AMC_FOS_SXT_NIF (16.7%)	CRO_CIP_AMC_FOS_SXT_NIF (16.7%)
Below Wetland (*n* = 29)	15 (51.7)	CRO_TET_CIP_NIF (18.8%)	CRO_TET_CIP_AMC_SXT_NIF (9.1%)
Downstream (*n* = 42)	15 (35.7)	CRO_CIP_NIF (17.9%)	CRO_CIP_AMC_FOS_SXT_NIF (2.8%)

**^1^** CIP, Ciprofloxacin; NIF, Nitrofurantoin; CRO, Ceftriaxone; TET, Tetracycline; AMC, Amoxicillin/Clavulanic acid; COL, Colistin; SXT, Trimethoprim-Sulfamethoxazole; FOS, Fosfomycin.

## Data Availability

The original data presented in the study are openly available in Harvard Dataverse. https://doi.org/10.7910/DVN/DEHMCT (accessed on 20 September 2024).
